# Short-term nonpressor angiotensin II infusion stimulates sodium transporters in proximal tubule and distal nephron

**DOI:** 10.14814/phy2.12496

**Published:** 2015-09-07

**Authors:** Mien T X Nguyen, Jiyang Han, Donna L Ralph, Luciana C Veiras, Alicia A McDonough

**Affiliations:** Department of Cell and Neurobiology, Keck School of Medicine, University of Southern CaliforniaLos Angeles, California, USA

**Keywords:** ENaC, intrarenal RAS, NCC, NHE3, NKCC2, SPAK

## Abstract

In Sprague Dawley rats, 2-week angiotensin II (AngII) infusion increases Na^+^ transporter abundance and activation from cortical thick ascending loop of Henle (TALH) to medullary collecting duct (CD) and raises blood pressure associated with a pressure natriuresis, accompanied by depressed Na^+^ transporter abundance and activation from proximal tubule (PT) through medullary TALH. This study tests the hypothesis that early during AngII infusion, before blood pressure raises, Na^+^ transporters’ abundance and activation increase all along the nephron. Male Sprague Dawley rats were infused via osmotic minipumps with a subpressor dose of AngII (200 ng/kg/min) or vehicle for 3 days. Overnight urine was collected in metabolic cages and sodium transporters’ abundance and phosphorylation were determined by immunoblotting homogenates of renal cortex and medulla. There were no significant differences in body weight gain, overnight urine volume, urinary Na^+^ and K^+^ excretion, or rate of excretion of a saline challenge between AngII and vehicle infused rats. The 3-day nonpressor AngII infusion significantly increased the abundance of PT Na^+^/H^+^ exchanger 3 (NHE3), cortical TALH Na-K-2Cl cotransporter 2 (NKCC2), distal convoluted tubule (DCT) Na-Cl cotransporter (NCC), and cortical CD ENaC subunits. Additionally, phosphorylation of cortical NKCC2, NCC, and STE20/SPS1-related proline–alanine-rich kinase (SPAK) were increased; medullary NKCC2 and SPAK were not altered. In conclusion, 3-day AngII infusion provokes PT NHE3 accumulation as well as NKCC2, NCC, and SPAK accumulation and activation in a prehypertensive phase before evidence for intrarenal angiotensinogen accumulation.

## Introduction

The renin–angiotensin system (RAS) affects sodium balance, extracellular fluid volume, and renal and vascular resistance. RAS blockers, including angiotensin-converting enzyme (ACE) inhibitors and angiotensin II (AngII) receptor blockers, have been widely used to reduce blood pressure in hypertensive subjects. How AngII regulates Na^+^ transport at the molecular levels has been a topic of research in many groups including our own. Acute AngII infusion (20 min) in anesthetized rats, without a rise in blood pressure, provokes PT NHE3 and Na^+^-P_i_ cotransporter 2 (NaPi2) redistribution into the microvilli where they likely contribute to a rapid increase in PT salt and water reabsorption (Riquier-Brison et al. 2006); acute AngII also provokes trafficking of DCT NCC from subapical vesicles to the plasma membrane (Sandberg et al. 2007) without an increase in NCC phosphorylation (Lee et al. 2013). In contrast, acute ACE inhibition by captopril redistributes NHE3 from the body to the base of the microvilli and NaPi2 from villi into a subapical vesicular compartment (Leong et al. 2006), and provokes acute NCC trafficking out of the plasma membrane (Sandberg et al. 2007). In these studies, acute AngII stimulation or inhibition regulates Na^+^ transport via redistribution of transporters, rather than changing their total pool size or covalent modification by phosphorylation.

Chronic AngII infusion is a well-studied model of experimental hypertension. Two weeks of AngII infusion provokes vasoconstriction and antinatriuresis which raises blood pressure, the error signal to drive pressure natriuresis, which matches Na^+^ output to Na^+^ input at the expense of elevated blood pressure (McDonough, 2010; Nguyen et al. 2013). When we profiled sodium transporter protein expression, we discovered that AngII infusion decreases Na^+^ transporter abundance and activation from proximal tubule through medullary TALH and increases Na^+^ transporter abundance and activation from the cortical TALH through medullary CD (Nguyen et al. 2013). Similar findings are evident in our analyses of transporters in AngII-infused mice (Gurley et al. 2011; Kamat et al. 2015). Importantly, the activation of TALH and DCT sodium transporters during chronic AngII infusion is blocked (and hypertension blunted) in mice that lack the ability to generate intrarenal AngII (Gonzalez-Villalobos et al. 2013) and in mice that lack interferon gamma production (Kamat et al. 2015) demonstrating that the infused AngII, per se, is not sufficient to stimulate these transporters during chronic AngII hypertension.

These studies have informed us that the responses of sodium transporters to *acute* AngII are distinct from the responses to *chronic* AngII: acutely, both proximal tubule NHE3 and distal tubule NCC redistribute into domains of apical membranes where they are active (without changes in abundance or phosphorylation); during chronic AngII infusion, with accompanying hypertension, the abundance of proximal NHE3 in the apical membrane is depressed, whereas abundance and phosphorylation of distal NCC in apical membranes is increased. In contrast, acutely raising blood pressure by vasoconstriction causes NHE3 to redistribute to the base of the microvilli where its activity is depressed (Zhang et al. 1998; Yip et al. 2000; Brasen et al. 2014) and causes NCC to redistribute from apical to subapical vesicles (Lee et al. 2009), accompanied by rapid and pronounced natriuresis and diuresis.

This study aimed to investigate the responses of transporters during the early phase of chronic AngII infusion, before hypertension, inflammation and activation of intrarenal AngII production are expected to ensue. The results indicate that short-term low-dose AngII infusion (200 ng/kg/min for 3 days) creates a prehypertensive state in which the abundance of proximal NHE3, cortical NKCC2, distal NCC, and cortical CD ENaC subunits, as well as phosphorylation of cortical NKCC2, NCC, and SPAK, are significantly increased, independent of accumulation of local AngII precursors. These findings contribute to our understanding of how hypertension develops during AngII-dependent hypertension at the molecular level and set the stage for addressing how natriuretic and antinatriuretic stimuli interact.

## Materials and Methods

### Animal protocols

All animal procedures were approved by the Institutional Animal Care and Use Committee of the Keck School of Medicine of the University of Southern California and were conducted in accordance with the National Institutes of Health Guide for the Care and Use of Laboratory Animals. Male Sprague Dawley rats (240–260 g body weight), obtained from Harlan Laboratories (San Diego, CA), were anesthetized intramuscularly with 200 *μ*L of ketamine and xylazine (1:1 volume ratio) and randomized to two groups. Osmotic minipumps (Alzet, model 2002, Cupertino, CA) containing AngII (200 ng/kg/min; Sigma) or vehicle (5% acetic acid, “control”). Infusion was continued for 3 days during which the rats had free access to normal vivarium diet.

### Physiological measurements

Rats were placed in metabolic cages overnight (16 h) for urine collection both before minipump implantation (day 1) and before sacrifice (day 2). Urine volumes were measured with graduated cylinders, urinary [Na^+^] and [K^+^] were measured by flame photometry (Radiometer FLM3). Urinary angiotensinogen was assessed by immunoblot in a constant fraction (0.02%) of the overnight urine volume.

After 3 days of AngII or vehicle infusion, rats were anesthetized intramuscularly with ketamine/xylazine, kidneys were excised rapidly, chilled in iced saline, and cortex and medulla dissected for homogenization. Blood samples were collected by cardiac puncture, hearts removed, flushed with PBS, blotted, and weighed. Plasma was prepared from blood by centrifugation for electrolyte measurements.

### Homogenate preparation and quantitative immunoblotting

Kidney cortex and medulla were rapidly dissected over ice, diced and homogenized as described previously (Nguyen et al. 2013), and quick-frozen in single use aliquots in liquid N_2_; protein concentration was determined by BCA assay (Pierce Thermo, Rockford, IL). Cortical and medullary homogenates were denatured in SDS-PAGE sample buffer for 20 min at 60°C (Nguyen et al. 2013). To verify uniform protein concentration, 10 *μ*g of protein from each sample was resolved by SDS-PAGE, stained with Coomassie blue, and multiple random bands quantified and determined to be uniform (if not, protein reassessed and gel rerun) as described previously (McDonough et al. 2015). For immunoblot, each sample was run at both one and one-half amounts to verify linearity of the detection system on each immunoblot. Antibodies used in this study, dilutions, and vendors are catalogued in Table 1. Signals were detected with Odyssey Infrared Imaging System (Li-COR) and quantified with accompanying software. Arbitrary density units collected were normalized to mean intensity of control group, defined as 1.0. Since the samples were run twice (at 1 and ½), the normalized values were averaged and mean values compiled for statistical analysis.

**Table 1:** Immunoblot antibody details

Antibody target	Primary Ab supplier	Ab host	Dilution	Incubation time/temp
NHE3 total	McDonough	Rb	1:2000	O/N 4°C
NHE3pS552	Santa Cruz	Mu	1:1000	2 h RT
NKCC total	C. Lytle (UCR)	Mu	1:6000	O/N 4°C
NKCCpT96pT101	Forbush (Yale)	Rb	1:2000	2 h RT
NCC total	McDonough	Rb	1:5000	O/N 4°C
NCCpT53	Loffing (Zurich)	Rb	1:5000	2 h RT
NCCpS71	Loffing (Zurich)	Rb	1:5000	2 h RT
NCCpS89	Loffing (Zurich)	Rb	1:5000	2 h RT
*α*-ENaC	Loffing (Zurich)	Rb	1:5000	2 h RT
*β*-ENaC	Loffing (Zurich)	Rb	1:15,000	2 h RT
*γ*-ENAC	Palmer	Rb	1:2500	O/N 4°C
SPAK total	Delpire (Vanderbilt)	Rb	1:3000	O/N 4°C
SPAKpS373	DSTT (Dundee)	Sheep	6 *μ*g/25 mL	2 h RT
Angiotensinogen	Sernia (Queensland)	Sheep	1:5000	2 h RT
Hsp70	Cayman	Mu	1:5000	O/N 4°C

Same conditions for cortex and medulla.

Rb, rabbit; Mu, mouse; O/N, overnight (16 h); R/T, room temperature.

### Saline challenge test

A separate set of four rats at baseline was acutely anesthetized with isoflurane then injected i.p. with a volume of isotonic saline (warmed to 37°C) equivalent to 7% of their body weight then placed immediately in metabolic cages for urine collection over 4 h without access to food. The same four rats were subsequently infused with AngII for 3 days, as described earlier, and the saline challenge was repeated. Results are expressed as mmol Na^+^ excreted/4 h/kg body weight, at both before and after the AngII infusion.

### Statistical analysis

Differences in physiological parameters and in protein total abundance and phosphorylation were assessed by unpaired two-tailed Student's *t*-test. Data were expressed as means ± SEM. Differences were regarded significant at *P *<* *0.05.

## Results

### Short-term nonpressor AngII did not alter physiological parameters

Baseline body weight, urine volume (UV), urinary Na^+^ and K^+^ excretion (U_Na_V and U_K_V, respectively) were, as expected, indistinguishable in the control versus treated groups before treatment (data not shown) and these parameters were not significantly altered by 3 days AngII (Table 2). Previous reports have shown that during the first 3 days of AngII infusion, even at a higher infusion doses, blood pressure is not significantly elevated compared to sham-operated animals (Zou et al. 1996; Kawada et al. 2002; Gonzalez et al. 2014). Therefore, in our study, hypertension is not expected, and there was no pressure diuresis or natriuresis, no cardiac hypertrophy, and no change in hematocrit, thus, no evidence for acute volume expansion or hypertension, also no AngII associated reduced rate of weight gain, nor change in hematocrit, all previously reported by us and others during weeks long infusion of AngII (Crowley et al. 2006; Ortiz et al. 2007; Semprun-Prieto et al. 2011; Nguyen et al. 2013). Finally, AngII infusion did not significantly change plasma [Na^+^] or [K^+^] indicating that electrolyte balance was maintained.

**Table 2:** Three day subpressor AngII infusion (200 ng/kg/min, 3 days) does not significantly alter physiological parameters

Physiological parameters	Control (*n *=* *7)	AngII (*n *=* *7)
Hematocrit	0.45 ± 0.01	0.45 ± 0.01
Plasma Na^+^, mmol/L	135.6 ± 2.1	138.4 ± 1.0
Plasma K^+^, mmol/L	4.5 ± 0.1	4.7 ± 0.1
Body wt gain per day, g	4.8 ± 0.5	4.8 ± 0.5
Heart wt/100 g body wt	0.29 ± 0.01	0.31 ± 0.005
Kidney wt/100 g body wt	0.44 ± 0.01	0.43 ± 0.01
Baseline overnight UV, mL	12.3 ± 0.9	11.3 ± 1.0
Baseline overnight U_Na_V, mmol	1.9 ± 0.1	1.8 ± 0.1
Baseline overnight U_K_V, mmol	3.2 ± 0.1	3.1 ± 0.1
Final overnight UV, mL	13.3 ± 1.0	14.1 ± 1.3
Final overnight U_Na_V, mmol	2.1 ± 0.1	2.1 ± 0.1
Final overnight U_K_V, mmol	3.5 ± 0.1	3.3 ± 0.1

“Baseline” urine was collected 16 h overnight before minipump implantation at day 1.

“Final” urine was collected overnight before euthanizing at day 3.

UV, urine volume; UNaV, urinary Na^+^ excretion; UKV, urinary K^+^ excretion.

**Figure 1:**
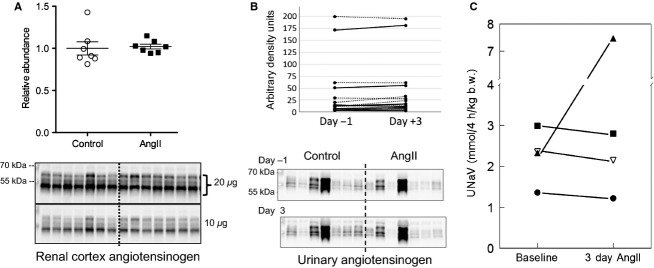
Effects of 3-day subpressor AngII infusion on (A) renal cortical angiotensinogen expression, (B) urinary angiotensinogen excretion, and (C) natriuretic response to saline challenge. Immunoblots of cortical (A) and urinary (B) angiotensinogen of rats infused with either vehicle (Control o) or AngII (_▀_) (*n *= 7). (A) 20 and 10 *μ*g renal cortical protein loaded per lane. Relative abundance displayed as individual records with means ± SEM, (B) 0.02% of overnight urine volume before minipump implantation (day 1) and before sacrifice (day +3) displayed as paired arbitrary density units connected by either dotted lines (controls) or solid lines (AngII group); (C) at baseline, then repeated after 3 days of AngII infusion, rats were challenged with an i.p. bolus of saline equivalent of 7% of their body weight and placed in metabolic cages for urine collection (*n *= 4). Results are displayed as paired urinary Na^+^ excretion (UNaV) collected over 4 h, connected by solid lines.

During AngII hypertension, the intrarenal RAS is activated evident as increased renal content of angiotensinogen, ACE, and AngII (Navar et al. 2011). Urinary angiotensinogen excretion is also increased, likely reflecting increases in both intrarenal production and filtration from plasma across a compromised filtration barrier (Kobori et al. 2002; Matsusaka et al. 2012). Our previous studies in rats verified that 2 week AngII infusion (400 ng/kg/min) increased urinary angiotensinogen more than 10-fold (Nguyen et al. 2013). In contrast, 3 days of AngII infusion did not raise renal cortical angiotensinogen abundance (Fig. 1A), nor increase urinary angiotensinogen excretion (Fig. 1B) indicating that intrarenal RAS is not significantly stimulated at this early stage, and that glomerular permeability to angiotensinogen is not increased. Interestingly, in both control and AngII-infused groups there were single rats that excreted very high levels of angiotensinogen at both baseline (day 1) and after 3 days AngII, independent of renal cortical angiotensinogen abundance.

To evaluate the effect of 3 days of AngII infusion on overall Na^+^ handling by the kidney, we challenged rats with an i.p. bolus of warmed saline equivalent to 7% of their body weight at baseline and again after 3 days of AngII infusion. Isotonic volume expansion has been reported to raise renal plasma flow and GFR while also decreasing renal O_2_ consumption indicative of renal tubular transport inhibition (Weinstein & Szyjewicz, 1976). The assumption is that while the rats will all eventually excrete the entire saline load, the rate of excretion will be a function of sodium transporter activation along the nephron, that is, slower excretion in rats with more Na^+^ transporter activation after AngII infusion. Since 7% of their body weight was roughly 20 mL, the volume of isotonic saline injected contained 3.1 mEq Na^+^. Results (Fig. 1C) are displayed as paired urinary Na^+^ excretion (UNaV) collected over 4 h, connected by solid lines. Three of the rats exhibited a consistent 10% depression in the UNaV after AngII, supporting the idea that AngII stimulation of transporters counteracts the volume expansion diuresis. A fourth rat unexpectedly exhibited a UNaV that was more than double the amount of Na^+^ injected; half of this Na^+^ was excreted during the first 2 h suggesting that there was a large amount of Na^+^ in the bladder at the outset, a drawback of this procedure. These findings, coupled to those in Table 2, indicate that after 3 days of a subpressor dose of AngII, rats remain in a prehypertensive state in which electrolyte homeostasis is maintained, yet there is evidence for slower excretion of a saline challenge.

### Effects of short-term low-dose AngII on renal transporters expression along the nephron

**Figure 2:**
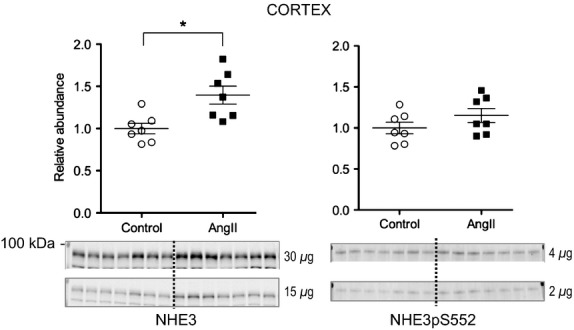
Short-term low-dose AngII infusion increases cortical NHE3 abundance but not NHE3pS552 abundance. Immunoblots of NHE3 total (30 and 15 *μ*g/lane) and NHE3pS552 (4 and 2 *μ*g/lane) in renal cortex homogenates of rats infused with either vehicle (Control, ○) or AngII (_▀_) (*n *= 7). Relative abundance displayed as individual records with means ± SEM. *P < 0.05.

NHE3 is expressed in the apical membranes of epithelial cells along the entire proximal tubule (PT) as well as in the thick ascending loop of Henle (TALH). We previously reported that 2 weeks AngII hypertension significantly depressed NHE3 abundance in both locations, associated with diuretic and natriuretic responses (Nguyen et al. 2013). In contrast, the current study shows that 3-day subpressor infusion of AngII significantly increases cortical NHE3 abundance to 1.40 ± 0.11 of control (Fig. 2A). Abundance of NHE3 phosphorylated at serine 552 (NHE3pS552), a marker for its transit to the base of the microvilli and inactivation (Kocinsky et al. 2005; Kocinsky et al. 2007), was unchanged (Fig. 2B), yet the overall ratio of NHE3pS552/NHE3, a marker of the fraction of the NHE3 in the inactive domain, was marginally decreased by 15% (*P *=* *0.051) consistent with net AngII activation of the greater pool of NHE3 at day 3.

**Figure 3:**
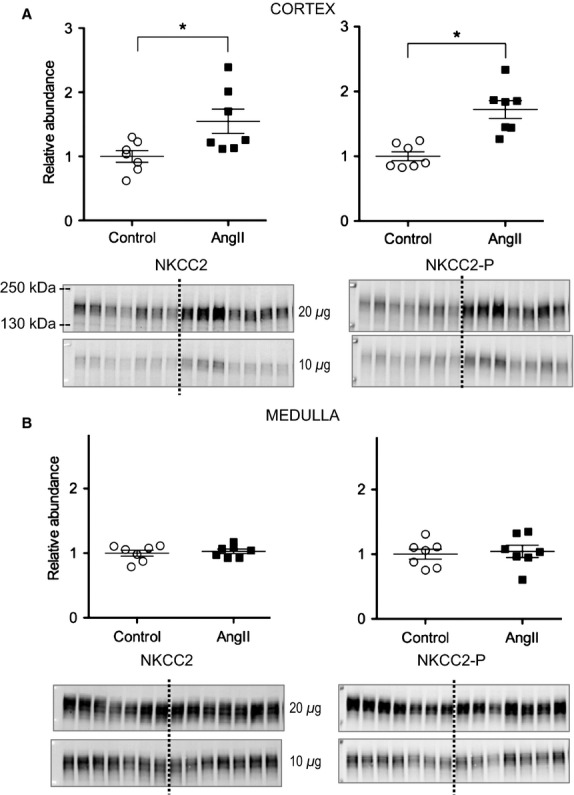
Short-term low-dose AngII infusion increases cortical but not medullary NKCC abundance and phosphorylation. Immunoblots of NKCC total and NKCC2pT96T101 (20 and 10 *μ*g/lane) in homogenates of renal cortex (A) and medulla (B) of rats infused with either vehicle (Control, ○) or AngII (_▀_) (*n *= 7). Relative abundance displayed as individual records with means ± SEM. *P < 0.05.

Moving down the nephron, the main apical transporter in the medullary and cortical loop of Henle is the loop diuretic sensitive Na^+^-K^+^-2Cl^−^ cotransporter (NKCC2). We previously reported that 2 week AngII hypertension significantly depressed medullary NKCC2 while significantly stimulating cortical NKCC2 abundance. In this study, 3 days subpressor AngII did not change medullary NKCC2 nor its phosphorylation at threonine residues 96 and 101 (NKCC2-P), a surrogate marker of activation, however, the 3-day AngII treatment significantly increased cortical TALH NKCC2 and NKCC2-P abundance by 1.55 ± 0.19 and 1.72 ± 0.14 fold over control levels, respectively, evidence for AngII stimulation of cortical, not medullary, NKCC2 during early phase of AngII infusion (Fig. 3).

**Figure 4:**
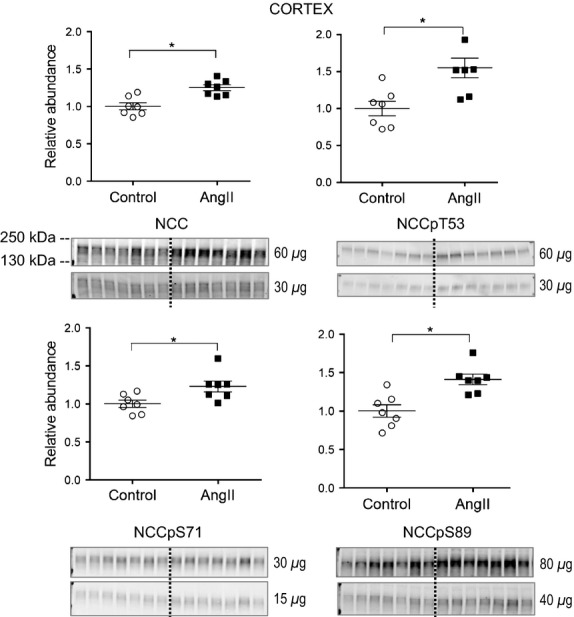
Short-term low-dose AngII infusion increases cortical NCC abundance and phosphorylation. Immunoblots of NCC total and NCCpT53 (60 and 30 *μ*g/lane), NCCpS71 (30 and 15 *μ*g/lane), and NCCpS89 (80 and 40 *μ*g/lane) in homogenates of renal cortex of rats infused with either vehicle (Control, ○) or AngII (_▀_) (*n *=* *7). Relative abundance displayed as individual records with means ± SEM. *P < 0.05.

Multiple laboratories, including our own, have provided evidence that the angiotensin II regulates distal convoluted tubule NCC acutely by increasing apical distribution via trafficking to the apical membrane (Sandberg et al. 2007; Ko et al. 2015), and chronically by increasing its abundance and phosphorylation (Gamba, 2012; Nguyen et al. 2013). NCC is phosphorylated at multiple sites and phosphorylation is associated with its activation (Richardson et al. 2011). Additionally, NCC-P is located exclusively in the apical membrane (Pedersen et al. 2010; Lee et al. 2013). After 3 days of AngII infusion, abundance of NCC total, NCC phosphorylated at threonine residue 53 (NCCpT53), at serine residue 71 (NCCpS71), and at serine residue 89 (NCCpS89) significantly increase (to 1.25 ± 0.04, 1.55 ± 0.13, 1.23 ± 0.07, and 1.41 ± 0.07 of control, respectively) (Fig. 4), all evidence of AngII stimulation of DCT NCC after 3 days.

**Figure 5:**
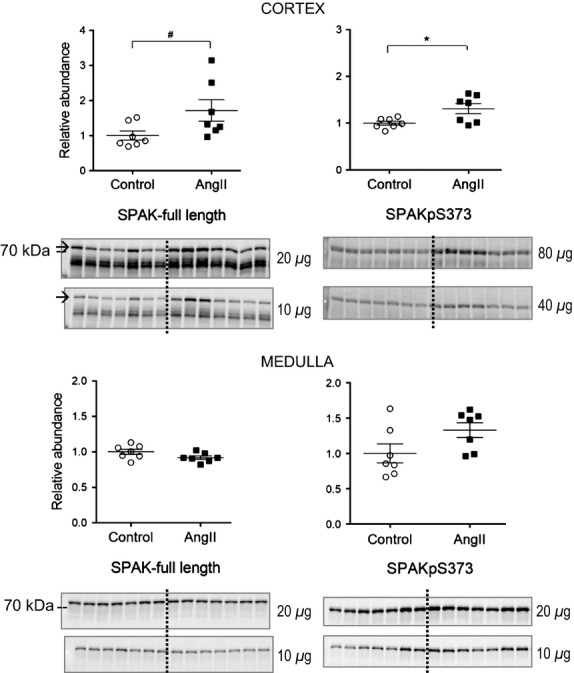
Short-term low-dose AngII infusion increases cortical but not medullary SPAK abundance and phosphorylation. Immunoblots of SPAK total (20 and 10 *μ*g/lane) and SPAKpS373 (80 and 40 *μ*g/lane in cortex, 20 and 10 *μ*g/lane in medulla) in homogenates of renal cortex (A) and medulla (B) of rats infused with either vehicle (Control, ○) or AngII (_▀_) (*n *=* *7). Relative abundance displayed as individual records with means ± SEM. *P < 0.05, ^#^*P = *0.05.

The STE20/SPS1-related proline–alanine-rich kinase (SPAK) is phosphorylated and activated by chronic AngII treatment and its substrates include NKCC2 and NCC (Richardson et al. 2008; Richardson et al. 2011; Castaneda-Bueno & Gamba, 2012; van der Lubbe et al. 2013). SPAK phosphorylated at serine 373 (SPAKpS373) is an indicator of kinase activation. We have previously reported that during 2-week AngII infusion in rats, SPAKpS373 is increased in cortex and decreased in medulla, analogous to the pattern of NKCC2 regulation. After 3-day AngII infusion in the current study, SPAKpS373 mean abundance increased to 1.31 ± 0.11 fold over control in cortex (*P *=* *0.03) and tended to increase to 1.33 ± 0.10 fold over control in medulla (*P *=* *0.08) (Fig. 5); full length SPAK abundance in cortex also tended to increase 1.72 ± 0.31 fold (*P *=* *0.05) and was unchanged in the medulla (0.92 ± 0.02) after 3 days AngII versus vehicle treated (Fig. 5). These data provide evidence for stimulation of cortical SPAK, and no evidence for depressed medullary SPAK early during AngII infusion.

**Figure 6:**
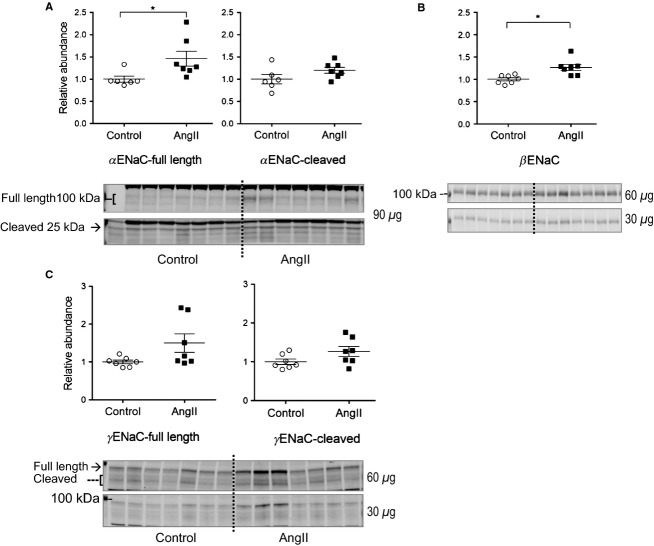
Short-term low-dose AngII infusion increases cortical ENaC abundance. Immunoblots of ENaC subunits in cortical homogenates of renal cortex of rats infused with either vehicle (Control, ○) or AngII (_▀_) (*n *=* *7). (A) *α* ENaC full length and cleaved forms indicated (90 *μ*g/lane shown). (B) *β* ENaC (60 and 30 *μ*g/lane). (C) *γ* ENaC full length and cleaved forms indicated (60 and 30 *μ*g/lane). Relative abundance displayed as individual records with means ± SEM. *P < 0.05.

The epithelial Na channel of the late DCT and collecting duct is composed of *α*, *β*, and *γ* subunits. Activity is regulated by changing abundance of the heteromer in the membrane as well as by proteolytic cleavage of the *α* and *γ* subunits (Rossier et al. 2015). Our work in rats and mice demonstrate increased abundance and cleavage of ENaC during 2 weeks AngII infusion (Gonzalez-Villalobos et al. 2013; Nguyen et al. 2013; Kamat et al. 2015). As summarized in Figure 6, 3 days of AngII infusion significantly increased the abundance of full length *α* and *β* subunits by 1.46 ± 0.17 and 1.27 ± 0.07 fold over control levels, respectively; full length *γ*, and cleavage of *α* and *γ* subunits were not yet significantly increased at this early phase of AngII infusion.

## Discussion

Our previous analyses of chronic AngII infusion with accompanying hypertension demonstrated that a new homeostatic state was achieved that balanced AngII stimulation of Na^+^ transporters from the cortical TALH to the collecting duct with hypertension-driven compensatory inhibition of Na^+^ transporters along the proximal tubule and medullary TALH, resulting in effective circulating volume homeostasis at normal levels. In this study, we asked whether short-term subpressor low-dose AngII without counteracting hypertension would stimulate proximal and TALH transporters including NHE3 and NKCC2, and asked whether this low-dose AngII would also stimulate distal transporters, including cortical NKCC, NCC, and ENaC without the augmented intrarenal RAS activation observed during 2-week AngII infusion. From the physiological parameters measured (Table 2 and Fig. 1), we conclude that the rats are in a prehypertension, preinjury state in which fluid and electrolyte balance is maintained and conclude that the rats display no evidence of hypertension, natriuresis, or diuresis, all evident at 2 weeks infusion of a higher dose of AngII (Nguyen et al. 2013). In an earlier study, Zou and Navar (Zou et al. 1996) infused a dose of AngII equivalent to that used in this study over a time course. At day 3, plasma [AngII] doubled from 50 to 100 fmol/mL, intrarenal AngII was unchanged before day 10 and blood pressure did not increase before day 6.

**Figure 7:**
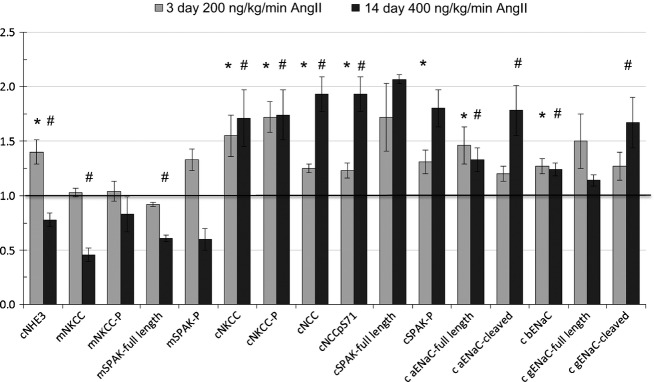
Renal transporter profiles of Sprague Dawley rats infused with 3 day versus 2 week subpressor regimes of angiotensin II. Data from subpressor AngII treatment (200 ng AngII/kg/min for 3 days, light gray bars, *n *=* *7) are summarized from Figures 6 and normalized to relative abundance in control rats infused with saline (defined as =1, indicated with a horizontal line at 1.). Data from longer AngII treatment (400 ng/kg/min × 12 days, dark gray bars, *n* = 8) are summarized from Nguyen et al. (2014) and also normalized to saline-infused controls. *Note*: only one phosphorylated form of NCC is included as the other forms change in the same directions. *P < 0.05 versus saline infused rats in 3-day AngII study, ^#^*P* < 0.05 versus saline infused rats in 2-week AngII study.

Figure 7 compares the transporter profiles obtained in the current study in response to 3 days of AngII (200 ng/kg/min) to the profiles obtained in a previous study of rats treated for 2 weeks with AngII (400 ng/kg/min) conducted in the same laboratory by the same investigators (Nguyen et al. 2013). In both sets, the AngII-treated results are expressed relative to the mean relative abundance of sham-treated rats defined as 1.0 (indicated by a horizontal line at 1.0). The most striking finding confirms our hypothesis that NHE3 is stimulated after 3-day subpressor AngII infusion and suppressed, along with mNKCC and mSPAK, by longer term AngII infusion associated with hypertension, indicating that the hypertension literally overrides the influence of the infused AngII. We predict that this short-term subpressor AngII infusion retains NHE3 localization within the microvilli in addition to increasing its total pool size as NHE3 was localized to the body of the microvilli after 2 weeks, despite lower abundance (Nguyen et al. 2013). Li and Zhou examined the signaling responses to low dose nonpressor versus pressor dose AngII infusion in the proximal tubule. Interestingly, they reported that NHE3 phosphorylated at serine 552, a marker for inactive NHE3 at the base of the microvilli (Kocinsky et al. 2007), was increased after low-dose nonpressor treatment and decreased after 2-week pressor dose treatment. One potential explanation to reconcile their findings with ours is that the conditions they used to detect anti NHE3-P also detected total NHE3, which we observed to be increased with low-dose AngII and decreased during chronic AngII hypertension.

Proximal NHE3 regulation during AngII is complicated not only by the opposing forces of AngII and pressure natriuretic factors, but also by the reports of biphasic effects of AngII on proximal function measured during micropuncture and microperfusion in rodents: stimulation of NHE3 by [AngII] in physiologic range and inhibition of NHE3 by AngII above 10^−7^ mol/L (Harris & Navar, 1985). The biphasic nature of the AngII effect suggests the possibility that the depressed NHE3 abundance observed during the 2-week AngII infusion could be due to [AngII] elevated in the renal tubules, specifically above 10^−7^ mol/L. Wang, Navar, and Mitchell directly assayed proximal tubule fluid AngII concentration and discovered that [AngII] was 10-fold higher in tubular fluid than in plasma at baseline and further elevated during 2-week AngII infusion. However, concentrations only reached the nanomolar range, 100 times less than the doses reported to inhibit NHE3 (Wang et al. 2003). These findings are consistent with stimulatory concentrations of renal AngII at subpressor infusion doses (200–400 ng/kg/min) from 3 days to 2 weeks. These AngII doses are expected to stimulate sodium transporters, thus, impair pressure natriuresis and eventually provoke hypertension, renal injury, and intrarenal production of AngII (McDonough and Nguyen 2015).

Further along the nephron in the medulla, 3 days of AngII infusion did not alter medullary NKCC, SPAK, or their activated phosphorylated forms, while both medullary NKCC2 and SPAK were suppressed during 2-week AngII hypertension. These findings suggest that at this dose and time, medullary SPAK and/or NKCC2 are not direct or indirect targets of AngII signaling and, thus, are not likely to play a vital role in the development of AngII hypertension, rather, they appear key to the compensatory natriuretic adjustments after hypertension develops.

Moving along to the cortical TALH, 3 days AngII infusion significantly increased cortical NKCC, NKCC-P, NCC, NCC-P, SPAK, SPAK-P as well as ENaC *α* and *β* subunits, consistent with stimulation by the infused (rather than intrarenal produced) AngII, as intrarenal AngII synthesis and accumulation does not become significant until day 6 at this infusion rate (Zou et al. 1996). Elevated sodium transporters from the cTALH to the CD likely play a role in the ultimate development of hypertension seen at higher doses and longer infusion times. After 2 weeks of 400 ng/kg/min AngII infusion with accompanying hypertension, no further increases in NKCC, NKCC-P, ENaC *α* or *β* subunits were evident, however, there further increases in NCC, NCC-P, *α*ENaC cleavage, and *γ*ENaC cleavage were evident (Fig. 7) that could be attributed to intrarenal AngII production, aldosterone activation, or accompanying inflammation and injury. Interestingly, the responses of cortical NKCC2 are distinct from the responses of medullary NKCC2 at both the lower dose 3 days AngII and the higher dose 14 days AngII suggesting different signaling cascades are operating on NKCC2 in cortex versus medulla.

Tiwari and Ecelbarger previously evaluated sodium transporter abundance in normal adult mice by immunoblot after 7 days of infusion with 800 ng/kg/min AngII (Tiwari et al. 2009, 2010). Blood pressure rose to 160 mmHg in their model, and cortical NHE3, distal NCC, cleaved *γ*ENaC were reported to be increased and whole kidney NKCC reported to be lower in abundance in the AngII-treated group. The rise in NCC and ENaC and fall in NKCC2 are similar to the findings in Figure 7, while the increase in NHE3 abundance is inconsistent with our findings. Increased NHE3 during AngII infusion with hypertension is not consistent with mouse versus rat effects because we also observed lower NHE3 (Gurley et al. 2011) or no increase in NHE3 (Gonzalez-Villalobos et al. 2013; Kamat et al. 2015) in mouse studies in this laboratory during AngII hypertension.

In AngII-dependent hypertension, increased intrarenal angiotensinogen expression and urinary angiotensinogen excretion are indicators of both breakdown of the glomerular filtration barrier as well as activation of intrarenal angiotensinogen synthesis; subsequently, more AngII is produced and spilled over in the tubular fluid (Kobori et al. 2002; Navar et al. 2011). We have reported that accumulation of intrarenal AngII is essential to activate Na^+^ transporters in the distal nephron and exacerbates hypertension (Gonzalez-Villalobos et al. 2013). In the current study, we examined the activation of intrarenal RAS at the early phase of AngII infusion and demonstrated that both cortical angiotensinogen and urinary angiotensinogen excretion were not yet increased after 3-day AngII infusion (Fig. 1) indicating that intrarenal angiotensinogen is likely not to be activated at this early time point and consistent with previous studies (Zou et al. 1996). Additional factors have been shown to be required for the robust expression of proximal angiotensinogen and for urinary angiotensinogen excretion. For example, interleukin 6, the most highly secreted cytokine during AngII induction (Recinos et al. 2007), stimulates angiotensinogen expression in cultured human renal proximal tubular cell cultures through activation of STAT3 (Signal Transducer and Activation of Transcription factor), suggesting a role in proximal tubule in vivo (Satou et al. 2009). Another example is that urinary angiotensinogen is higher when AngII-infused rats are fed a high salt diet, and the increase is mediated by the NADPH oxidase pathway, and rescued by treating animals with an AT1R blocker (Lara et al. 2012). Thus, accumulation of reactive oxygen species (ROS) in renal tissue contributes to increased urinary angiotensinogen excretion, reinforcing the positive feedback loop between RAS activation of ROS and ROS activation of angiotensinogen.

Relevant to our study, Prieto et al. (Gonzalez et al. 2014) recently provided evidence for a transient increase in membrane bound prorenin receptor ((P)RR) in collecting ducts after 3 days of AngII (400 ng/kg/min) infusion at which point there was no significant increase in blood pressure. The increase in PRR led to a transient increase in COX2 protein and urinary PGE3 which the authors postulated may play a buffering role in counteracting the antinatriuretic and vasoconstrictive effects of AngII at this 3 day, prehypertensive time point. This buffering could also play a role in the excretion of the saline load at 3 days (Fig. 1C), that is, account for why it was not more blunted. By 14 days of AngII infusion, membrane bound (P)RR was reduced and COX-2 protein levels were not different from controls (Gonzalez et al. 2014).

The results of this study, coupled to that of the others discussed, are consistent with the following summary: at an early stage of low dose of AngII infusion, Na^+^ transporters in the proximal tubule and in the distal nephron, not the medullary loop of Henle, are activated directly by AngII. When AngII is sustained in the system for a longer period, hypertension ensues and pressure natriuresis is provoked and overrides the antinatriuresis of AngII via suppression of PT and medullary TALH Na^+^ transporters (McDonough and Nguyen, 2015). Additionally, a positive feedback loop generating more AngII in the tubular fluid via the intrarenal RAS system is stimulated and necessary to maintain the activation of transporters in the distal nephron (Gonzalez-Villalobos et al. 2013) which maintains and exacerbates the elevated Na^+^ reabsorption in this region and the necessity for elevated pressure to drive pressure natriuretic decreases in proximal and loop of Henle transport in order to match Na^+^ output to input.

## Conflict of Interest

None declared.
